# Point-of-Care Antigen Test for SARS-CoV-2 in Asymptomatic College Students

**DOI:** 10.3201/eid2710.210080

**Published:** 2021-10

**Authors:** Sarah C. Tinker, Christine M. Szablewski, Anastasia P. Litvintseva, Cherie Drenzek, Gary E. Voccio, Melissa A. Hunter, Stephen Briggs, Debbie E. Heida, Jennifer Folster, Patricia L. Shewmaker, Magdalena Medrzycki, Michael D. Bowen, Caitlin Bohannon, Dennis Bagarozzi, Marla Petway, Paul A. Rota, Wendi Kuhnert-Tallman, Natalie Thornburg, Jessica L. Prince-Guerra, Lisa C. Barrios, Azaibi Tamin, Jennifer L. Harcourt, Margaret A. Honein, CDC Sample Collection Team,, CDC Surge Laboratory Group

**Affiliations:** Centers for Disease Control and Prevention, Atlanta, Georgia, USA (S.C. Tinker, C.M. Szablewski, A.P. Litvintseva, J. Folster, P.L. Shewmaker, M. Medrzycki, M.D. Bowen, C. Bohannon, D. Bagarozzi Jr., M. Petway, P.A. Rota, W. Kuhnert-Tallman, N. Thornburg, J.L. Prince-Guerra, L.C. Barrios, A. Tamin, J.L. Harcourt, M.A. Honein);; Georgia Department of Public Health, Atlanta (C.M. Szablewski, C. Drenzek);; Georgia Department of Public Health Northwest Health District, Rome, Georgia, USA (G.E. Voccio, M.A. Hunter);; Berry College, Mount Berry, Georgia, USA (S. Briggs, D.E. Heida)

**Keywords:** COVID-19, SARS-CoV-2, institutions of higher education, adolescents, young adults, antigen testing, colleges, universities, asymptomatic, respiratory infections, severe acute respiratory syndrome coronavirus 2, 2019 novel coronavirus disease, coronavirus disease, zoonoses, viruses, coronaviruses

## Abstract

We used the BinaxNOW COVID-19 Ag Card to screen 1,540 asymptomatic college students for severe acute respiratory syndrome coronavirus 2 in a low-prevalence setting. Compared with reverse transcription PCR, BinaxNOW showed 20% overall sensitivity; among participants with culturable virus, sensitivity was 60%. BinaxNOW provides point-of-care screening but misses many infections.

Point-of-care antigen testing provides results more quickly than real-time reverse transcription PCR (rRT-PCR). In August 2020, the US Food and Drug Administration granted emergency use authorization to the BinaxNOW COVID-19 Ag Card (BinaxNOW; Abbott Laboratories, https://www.abbott.com) for the detection of severe acute respiratory syndrome 2 (SARS-CoV-2) infection in persons with signs or symptoms of coronavirus disease (COVID-19) (*1*). However, administrative discretion permits the screening of asymptomatic persons, thereby enabling the rapid identification and isolation of infectious persons (*2*). To assess the abilities of BinaxNOW to screen asymptomatic persons for SARS-CoV-2 in a low-prevalence setting, we compared the performance of BinaxNOW and rRT-PCR using paired samples collected from students at a residential college in the United States.

## The Study

During November 2020, the county in which the college is located reported 467 RT-PCR–positive cases/100,000 persons and a 13.7% positivity rate (*3*). The school instituted COVID-19 mitigation policies, including mask mandates, social distancing in classrooms, enhanced cleaning measures, limited campus access, and encouragement of small, mutually exclusive social bubbles. Most (87%) students lived on-campus, and the COVID-19 prevalence among students was 0.6%.

In total, 1,827 students were eligible for SARS-CoV-2 testing on campus, excluding 162 students who had tested positive during the previous 90 days. Students who reported signs or symptoms of COVID-19 in the school’s daily online tracking system were directed to the campus health center for testing. 

All students at the testing event, which was conducted over 4 days in November 2020, were asymptomatic. We obtained student demographic data from college records. This activity was reviewed by the institutional review boards of the Georgia Department of Public Health, Centers for Disease Control and Prevention (CDC; Atlanta, GA, USA), and college; the study was conducted in accordance with applicable federal law and CDC policy.

Project staff directed students to self-collect 2 anterior nasal swab samples by inserting 1 swab into each nostril and then switching the swabs to obtain sample secretions from the other nostril. We tested 1 swab immediately using BinaxNOW and sent the other swab to CDC for rRT-PCR.

We conducted BinaxNOW assays on-site in accordance with manufacturer instructions (*4*). Students received BinaxNOW results after 15–30 minutes. Those who tested positive were counseled to isolate for 10 days and interviewed for contact tracing.

Swab samples collected for rRT-PCR were stored using Remel R12587 viral transport media (Thermo Fisher Scientific, https://www.thermofisher.com) with cold packs; samples were transported daily to CDC and refrigerated at 4°C. We isolated nucleic acid from the specimens using the MagNA Pure 96 Instrument (Roche Molecular Systems, Inc., https://lifescience.roche.com) within 48 hours of collection, then analyzed the nucleic acid using the CDC Influenza SARS-CoV-2 (Flu SC2) Multiplex Assay (*5*). Results were reported as SARS-CoV-2–positive (cycle threshold [C_t_] <40 for the SARS-CoV-2 target), SARS-CoV-2–negative, or invalid (C_t_ value ≥40 for all viral targets and C_t_ >35 for human RNase P reference gene on repeat testing, according to the manufacturer’s guidelines).

We cultured residual frozen SARS-CoV-2–positive samples in 100 μL viral transport media. We limited dilution in Vero CCL-81 cells and monitored 96-well plates daily for cytopathic effects (J. Harcourt, unpub. data, https://www.biorxiv.org/content/10.1101/2020.03.02.972935v2). We extracted nucleic acid from the wells exhibiting cytopathic effects and confirmed the presence of SARS-CoV-2 by rRT-PCR. We considered a specimen to be culture-positive if the first viral passage had a C_t_ value >2 less than the clinical sample.

## Conclusions

In total, 1,540 asymptomatic students provided paired samples (Table). Forty (2.6%) samples tested positive by rRT-PCR; of these, 8 (20%) also tested positive by BinaxNOW. We did not observe any false-positive BinaxNOW results (100% specificity). Concordant samples had a lower median C_t_ value than discordant samples (21.9 vs. 34.9). Students received rRT-PCR results within 72 hours. No specimens tested positive for influenza A or B viruses. All 8 persons who tested positive by BinaxNOW and rRT-PCR later reported symptom onset. Among the 32 students who provided samples that tested negative by BinaxNOW and positive by rRT-PCR, 10 (31.3%) later reported symptom onset (median C_t_ 34.9), 16 (50.0%) later reported no symptoms (median C_t_ 35.1), and 6 (18.8%) did not report information on symptoms (median C_t_ 34.9).

**Table:** Characteristics of asymptomatic college students participating in study on point-of-care antigen test for severe acute respiratory syndrome coronavirus 2, 2020

Characteristic	No. (%)
Total	1,540
Age, y	
<18	362 (23.5)
19–20	733 (47.6)
21–22	418 (27.1)
23–24	18 (1.2)
>25	9 (0.6)
Sex	
M	549 (35.6)
F	991 (64.4)
Race/ethnicity*	
Non-Hispanic White	1,157 (75.4)
Non-Hispanic Black	112 (7.3)
Hispanic	133 (8.7)
Asian	43 (2.8)
Other†	89 (5.8)
Not available	6
Housing	
On-campus	1,379 (89.5)
Off-campus	161 (10.5)

*Percentages do not include the 6 persons whose race/ethnicity was not available. †Includes persons of >2 races.

We detected culturable virus in 5 (12.5%) samples that tested positive by rRT-PCR, including 3 (60%) that also tested positive by BinaxNOW (Figure). One person provided a sample (C_t_ 28.9) that tested negative by BinaxNOW but was culture-positive; symptoms later developed in this person, who tested positive by a different antigen test (BD Veritor System; Becton, Dickinson and Company, https://www.bd.com) the next day. Symptoms did not develop in the other person who provided a sample that tested negative by BinaxNOW and positive by culture (C_t_ 37.3).

**Figure:**
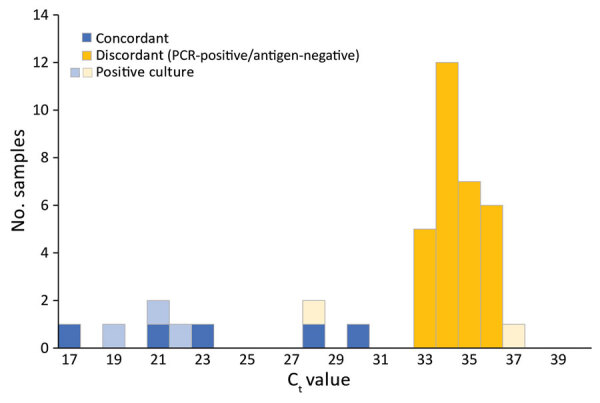
C_t_ values of severe acute respiratory syndrome coronavirus 2–positive samples tested by reverse transcription PCR, the BinaxNOW COVID-19 Ag Card (BinaxNOW; Abbott Laboratories, https://www.abbott.com), and viral culturing. C_t_, cycle threshold.

BinaxNOW provides rapid, point-of-care results; students received BinaxNOW results 3 days earlier than rRT-PCR results. However, BinaxNOW had low sensitivity, especially among persons with higher C_t_ values, which suggest lower viral load. BinaxNOW did not identify 32 persons who tested positive by rRT-PCR.

Our data are consistent with those of Prince-Guerra et al. (*6*), which found low overall BinaxNOW sensitivity (35.8%; 44/123) compared with RT-PCR among asymptomatic persons. Prince-Guerra et al. (*6*) found that concordant samples had a lower mean C_t_ value than discordant samples (22.5 vs. 33.9); we observed 88.9% (8/9) sensitivity among samples with C_t_ values <32. However, Prince-Guerra et al. (*6*) collected samples using disparate methods (nasopharyngeal swab for RT-PCR and anterior nasal swab for BinaxNOW), precluding direct comparison of samples. Our results are inconsistent with those of Pilarowski et al. (*7*), which showed 81.4% sensitivity among 102 persons who were asymptomatic or had symptom onset >1 week previously. We observed high specificity, consistent with results of both investigations (*6*,*7*). Unlike the community investigations of Prince-Guerra et al. (*6*) and Pilarowski et al. (*7*), in which testing was offered to persons who might have had specific reasons for seeking testing, our investigation was conducted in a closed, defined population, among persons with no known exposures or symptoms, providing more generalizable performance data for similar institutions.

CDC provided guidance on expanded screening testing of asymptomatic individuals to reduce spread of SARS-CoV-2 and for interpretation of antigen tests (*8*,*9*). Test performance among asymptomatic persons probably varies for different antigen tests. For example, an assessment of the Sofia SARS Antigen Fluorescent Immunoassay (Quidel Corporation, https://www.quidel.com) reported 41.2% sensitivity and 98.4% specificity among 871 asymptomatic college students (*10*).

Isolation of SARS-CoV-2 in cell culture demonstrates viral replication. However, because many factors affect the culture performance, lack of culturable virus does not necessarily indicate a lack of infectious virus. The presence of culturable virus in samples that test negative for SARS-CoV-2 antigens suggests that BinaxNOW does not identify some persons with infectious virus. However, the speed of BinaxNOW enabled the immediate identification of 8 SARS-CoV-2–positive persons, thereby limiting transmission that might have occurred during the additional 2 days that students waited for rRT-PCR results.

Although rRT-PCR tests remain standard for SARS-CoV-2 detection, point-of-care antigen tests such as BinaxNOW could increase access to serial screening, enabling the rapid identification and isolation of infectious persons. Because presymptomatic and asymptomatic persons can transmit SARS-CoV-2 (*11*), screening of asymptomatic persons is a key strategy for interrupting SARS-CoV-2 transmission. Although messaging must clearly communicate the low sensitivity of the test, positive results enable immediate public health action.

## References

[1] US Food and Drug Administration. BinaxNOW COVID-19 Ag Card emergency use authorization. 2020 [cited 2021 Jun 28]. https://www.fda.gov/media/147264/download

[2] Centers for Medicare and Medicaid Services. Updated CLIA SARS-CoV-2 molecular and antigen point of care test enforcement discretion. 2020 [cited 2021 Jun 28]. https://www.cms.gov/files/document/clia-sars-cov-2-point-care-test-enforcement-discretion.pdf

[3] Georgia Department of Public Health. Covid-19 daily status report, by county (Floyd County, November 16th). 2020 [cited 2021 Feb 28]. https://dph.georgia.gov/covid-19-daily-status-report

[4] US Food and Drug Administration. BinaxNOW COVID-19 Ag Card—instructions for use. 2020 [cited 2021 Jun 28]. https://www.fda.gov/media/141570/download

[5] US Food and Drug Administration. Influenza SARS-CoV-2 (Flu SC2) Multiplex Assay emergency use authorization. 2020 [cited 2021 Jun 28]. https://www.fda.gov/media/139744/download

[6] Prince-Guerra JL, Almendares O, Nolen LD, Gunn JKL, Dale AP, Buono SA, et al. Evaluation of Abbott BinaxNOW rapid antigen test for SARS-CoV-2 infection at two community-based testing sites—Pima County, Arizona, November 3–17, 2020 [Erratum in: MMWR Morb Mortal Wkly Rep. 2021;70:144]. MMWR Morb Mortal Wkly Rep. 2021;70:100–5. 10.15585/mmwr.mm7003e3 33476316 PMC7821766

[7] Pilarowski G, Marquez C, Rubio L, Peng J, Martinez J, Black D, et al. Field performance and public health response using the BinaxNOW rapid severe acute respiratory syndrome coronavirus 2 (SARS-CoV-2) antigen detection assay during community-based testing. Clin Infect Dis. 2020 Dec 26 [Epub ahead of print];ciaa1890. 10.1093/cid/ciaa1890 PMC7799223 33367619

[8] Centers for Disease Control and Prevention. Overview of testing for SARS-CoV-2 (COVID-19). 2021 Jun 14 [cited 2021 Jun 28]. https://www.cdc.gov/coronavirus/2019-ncov/hcp/testing-overview.html

[9] Centers for Disease Control and Prevention. Interim guidance for antigen testing for SARS-CoV-2. 2021 Jun 14 [cited 2021 Jun 28]. https://www.cdc.gov/coronavirus/2019-ncov/lab/resources/antigen-tests-guidelines.html

[10] Pray IW, Ford L, Cole D, Lee C, Bigoutee JP, Abedi G, et al. Performance of an antigen-based test for asymptomatic and symptomatic SARS-CoV-2 testing at two university campuses—Wisconsin, September–October 2020. MMWR Morb Mortal Wkly Rep. 2021;69:1642–7. 10.15585/mmwr.mm695152a3 33382679 PMC9191905

[11] Arons MM, Hatfield KM, Reddy SC, Kimball A, James A, Jacobs JR, et al.; Public Health–Seattle and King County and CDC COVID-19 Investigation Team. Presymptomatic SARS-CoV-2 infections and transmission in a skilled nursing facility. N Engl J Med. 2020;382:2081–90. 10.1056/NEJMoa2008457 32329971 PMC7200056

